# Surface Structure of Zirconia Implants: An Integrative Review Comparing Clinical Results with Preclinical and In Vitro Data

**DOI:** 10.3390/ma15103664

**Published:** 2022-05-20

**Authors:** Nadja Rohr, Blerta Hoda, Jens Fischer

**Affiliations:** Biomaterials and Technology, Clinic for Reconstructive Dentistry, University Center for Dental Medicine Basel, CH-4058 Basel, Switzerland; blerta.hoda@stud.unibas.ch (B.H.); jens.fischer@unibas.ch (J.F.)

**Keywords:** zirconia implants, surface structure, mean bone loss, bone-to-implant contact, osteoblasts, cell spreading, gene expression

## Abstract

Background: The purpose of this review was to analyze and correlate the findings for zirconia implants in clinical, preclinical and in vitro cell studies in relation to surface structure. Methods: Electronic searches were conducted to identify clinical, preclinical and in vitro cell studies on zirconia implant surfaces. The primary outcomes were mean bone loss (MBL) for clinical studies, bone-to-implant contact (BIC) and removal torque (RT) for preclinical studies and cell spreading, cell proliferation and gene expression for cell studies. The secondary outcomes included comparisons of data found for those surfaces that were investigated in all three study types. Results: From 986 screened titles, 40 studies were included for data extraction. In clinical studies, only micro-structured surfaces were investigated. The lowest MBL was reported for sandblasted and subsequently etched surfaces, followed by a sinter and slurry treatment and sandblasted surfaces. For BIC, no clear preference of one surface structure was observable, while RT was slightly higher for micro-structured than smooth surfaces. All cell studies showed that cell spreading and cytoskeletal formation were enhanced on smooth compared with micro-structured surfaces. Conclusions: No correlation was observed for the effect of surface structure of zirconia implants within the results of clinical, preclinical and in vitro cell studies, underlining the need for standardized procedures for human, animal and in vitro studies.

## 1. Introduction

Two-piece titanium dental implants with a roughened endosseous surface are frequently used to achieve fast and stable osseointegration [1,2,3,4]. The term osseointegration was introduced by Per-Ingvar Brånemark (Gothenburg, Sweden) for describing a direct structural and functional connection between living bone and the implant surface on a light microscopy level [5,6]. In the 1960s, Brånemark, working closely with the company Nobelpharma, considered a machined endosseous implant surface as appropriate. However, the group of André Schröder (Bern, Switzerland), cooperating with the company Straumann, introduced a rough surface that should stimulate the attachment of osteoblasts [7]. A wide range of different techniques has subsequently been developed by different implant manufacturers to equip the endosseous implant surface with unique features that would accelerate osseointegration [8,9,10]. The current consensus promotes an endosseous titanium implant surface with a moderately rough surface with an Sa of 1 to 2 µm for the highest osseointegration potential [11].

Hence, when zirconia implants were introduced, the endosseous surface was also structured in accordance with the consensus for titanium implants. Currently available zirconia implants are either made of 3 mol% yttria-stabilized tetragonal zirconia polycrystal (Y-TZP) or alumina-toughened zirconia (ATZ). The first zirconia implants on the market were sandblasted to create a rough surface. Currently available implants display moderately rough to rough features with smoothened edges created via sandblasting followed by acid etching, via laser ablation, additive sintering of small zirconia particles or injection molding [12,13,14,15]. Some implants are additionally heat-treated to retrieve the tetragonal phase and decelerate the aging procedure [13].

Clinical medium-term data are available for moderately rough zirconia implants [16,17,18,19]. For these implants, sandblasting followed by etching or optional heat-treatment or laser treatment is applied to create the moderately rough surface. Most studies report mean bone loss around the implant at different follow-ups based on radiographs. Survival or success rates are also commonly calculated.

Preclinical studies with zirconia implants have been conducted to compare mainly the bone-to-implant contact (BIC) or removal torque (RT) of zirconia with titanium implants [14]. The chosen animal models included rats, rabbits, sheep, pigs and monkeys [14,15]. A review of preclinical data with zirconia implant surfaces revealed that the BIC is rather dependent on the animal model than the surface roughness [14]. The osseointegration potential of zirconia was assessed in vitro by conducting experiments with a wide range of osteoblastic cells and stem cell lines [20,21]. Different parameters such as cell spreading, cell proliferation, cell viability or expression of a wide range of inflammation or osteogenic differentiation genes were evaluated. Reviews considering in vitro data of cell studies mainly compared zirconia with titanium surfaces [20,21], and only limited information is available about the influence of surface topography on cell behavior [22].

No review analyzed if the findings from in vitro cell studies are consistent with preclinical data and are consequently relevant for conducting clinical studies for the respective surface structures of zirconia. Hence, the purpose of this review was to analyze and correlate the findings for zirconia implants in clinical with preclinical and in vitro cell studies to evaluate the influence of the surface structure on the outcome.

## 2. Materials and Methods

This systematic review was conducted according to the Preferred Reporting Items for Systematic Review and Meta-Analysis Protocols (PRISMA-P [23]) statement using the Population, Intervention, Comparison and Outcome (PICO) method [24].

### 2.1. Focused Question

For the present review, the focused (PICO) question to be addressed was as follows: “In clinical, preclinical and in vitro studies, what are the outcomes (marginal bone loss, first bone-to-implant contact (BIC), removal torque (RT), cell behavior) of zirconia dental implant surfaces with regard to the surface structure?”

### 2.2. Search Strategy

A systematic electronic search of Medline via Pubmed and Embase was performed between June and August 2021. Articles in English and German were considered. The following terms and combinations were applied: ((“Dental implants” [MeSH] AND (“zirconium oxide” OR “yttria-stabilized tetragonal zirconia” OR “zirconia”)) OR “zirconia implant” OR “ceramic implant”) AND ((“osseointegration” [MeSH] OR “bone-implant-interface” [MeSH] OR “survival rate” [MeSH] OR “success rate” OR “marginal bone loss”) OR (“bone implant contact” OR “removal torque”) OR (“osteoblasts” OR “cell proliferation” OR “cell spreading” OR “gene expression”)). Additionally, recent systematic reviews [14,20,21,25,26,27] were screened for publications. Reference management software (Zotero, V 5.0.96.3) was used.

### 2.3. Inclusion Criteria

The inclusion criteria were defined as follows:

- Human trials, preclinical and in vitro osteoblastic cell studies investigating zirconia implant surfaces that were published between January 2000 and December 2021;
- Randomized clinical trials or cohort studies with at least 10 patients included [26];
- Details on the surface structure of zirconia and implant manufacturer are given;
- Reported details of marginal bone loss of clinical trials, BIC or RT of preclinical studies, osteoblastic cell behavior (spreading, proliferation, gene expression) for in vitro studies comparing two different zirconia surfaces.

### 2.4. Exclusion Criteria

Studies that did not meet the inclusion criteria were excluded from this work. Data from multiple publications on the same patient population were summarized, and the last published work was cited.

### 2.5. Selection of Studies

After elimination of duplicates, the reviewers (BH, NR) independently screened titles, abstracts and full texts meeting the selection criteria. Disagreements regarding the inclusion and exclusion of studies were resolved by discussion between the reviewers.

### 2.6. Data Extraction and Outcome Measures

Data extraction into Excel tables was independently performed for all included studies (BH, NR). From the included full-text articles, the following data were extracted: author(s), year of publication, number of included patients and implants, implant material (yttria-stabilized zirconia (Y-TZP)/alumina-toughened zirconia (ATZ), controls, implant design (1-piece/2-piece), specimen dimension, implant manufacturer, implant surface treatment, surface roughness, time period between implant placement and prosthetic treatment, type of restoration (single crown (SC)/fixed dental prostheses (FDP)), number of implant failures, observation period (months), implant survival (%), mean bone loss (MBL, mm), bone-to-implant contact (BIC, %), removal torque (RT, Ncm), animal, cell line, cell spreading, cell proliferation and gene expression.

Primary outcomes were MBL for clinical studies, BIC and RT for preclinical studies, and cell spreading, cell proliferation and gene expression for cell studies. Secondary outcomes included comparisons of data found for those surfaces that were investigated in all three study types.

### 2.7. Data Analysis

The mean MBL values from clinical studies of the same study population were collected for each follow-up. Data of studies using the same implant type were then pooled when data were extracted at the same follow-up. Further, data of implants with similar surface treatments were pooled at each follow-up. The BIC values and RT values of the included preclinical studies were listed for each intervention point. Values were further pooled by surface treatment and animal model for each intervention point. Minipig and pig were considered as species pig. Findings for cell spreading, cell proliferation and gene expression of in vitro cell studies were analyzed descriptively by comparing the results of the investigated surfaces within each study.

## 3. Results

**Figure 1:**
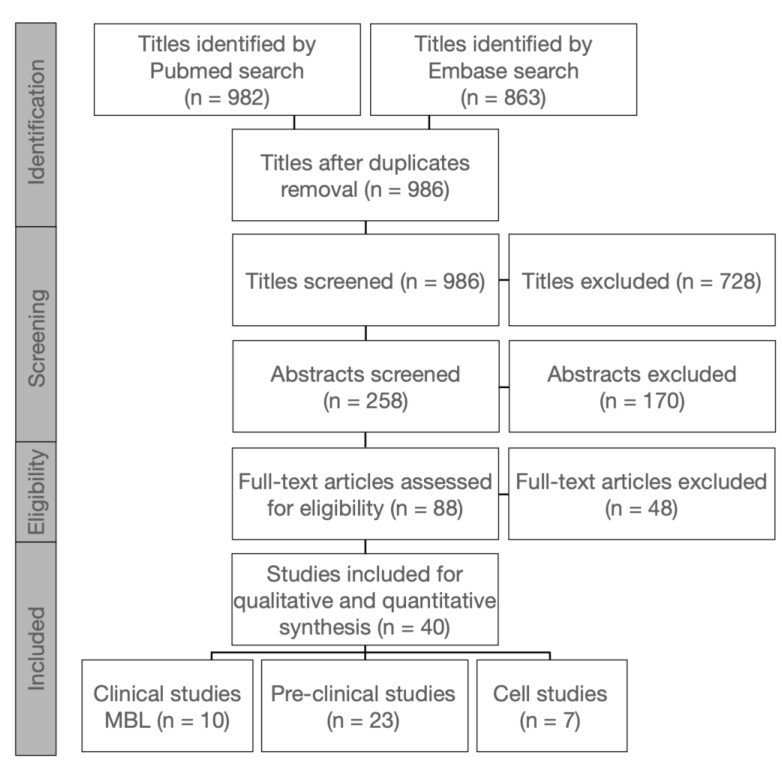
Search strategy and study selection process.

The electronic data base search and the respective results are displayed in Figure 1. For the clinical studies, only those displaying the latest results of the same study population are listed by the first authors’ name (n = 10). However, short-term data were extracted from previous studies of the respective population whenever data on MBL were missing in the latest reports.

**Figure 2:**
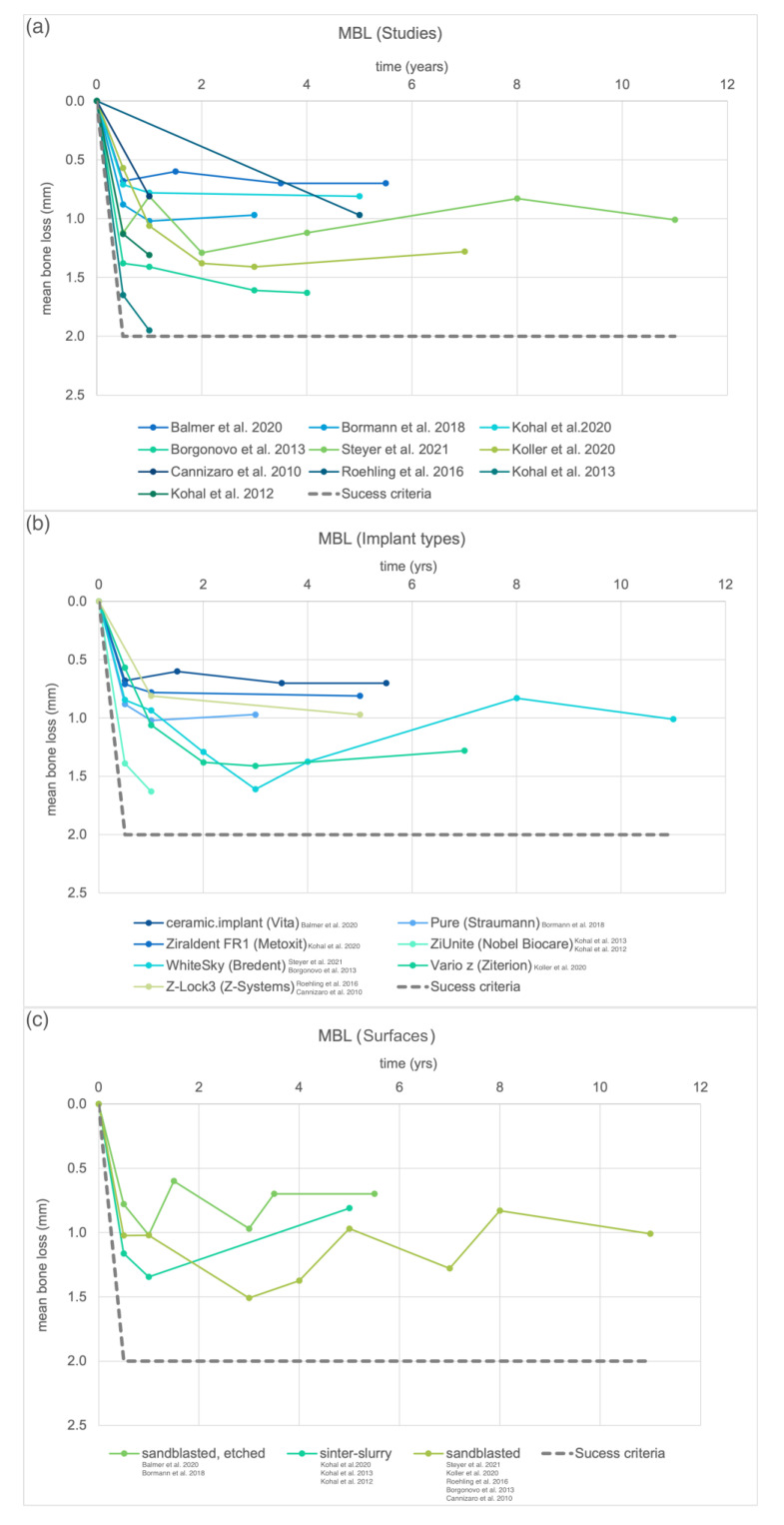
Mean bone loss (MBL) reported in clinical studies with zirconia implants at the respective follow-up. The threshold value included as success criteria was defined by Misch et al. 2008 [34]. (**a**) Mean bone loss of all included clinical trials, (**b**) mean bone loss pooled by implant type and (**c**) mean bone loss pooled by implant surface treatment.

Details on clinical studies are given in Table 1. Implants with sandblasted surfaces that were inserted in five of the studies [18,28,29,30,31] displayed survival rates between 77.3 and 100%. The survival rates of implants with sinter and slurry on the endosseous surface ranged from 94.3 to 98.2% [17,32,33]. The survival of sandblasted and etched surfaces that were used in two prospective studies ranged from 97.5 to 98.4% [16,19]. The MBL obtained at the respective follow-ups is displayed in Figure 2a. Following the consensus for titanium implants, an MBL of 2 mm at most was considered a threshold value for success [34]. The data of the same implant types were pooled in Figure 2b. The lowest MBL was observed with the implant ceramic.implant (Vita, Germany). The MBL was pooled by surface treatments in Figure 2c. Overall, the lowest MBL was reported for sandblasted and subsequently etched surfaces, followed by a sinter and slurry treatment and sandblasted surfaces. However, data of up to 11 years were included for sandblasted surfaces, while for the others, data between 3 and 5.5 years are currently published.

An overview of the animal studies is given in Table 2 (n = 23). Within those studies, the results of BIC and RT obtained within the same set-up were published separately for two preclinical studies [45,46,47,48]. The BIC was obtained in 19 and RT in 8 of the studies. Only one study compared the BIC of two different implant materials within one trial, however, with varying surface treatments [49]. The BIC values in studies directly comparing zirconia implants with different surfaces with the same set-up were either significantly higher [50], similar [51,52,53] or lower [54] for smoother surfaces. 

**Figure 3:**
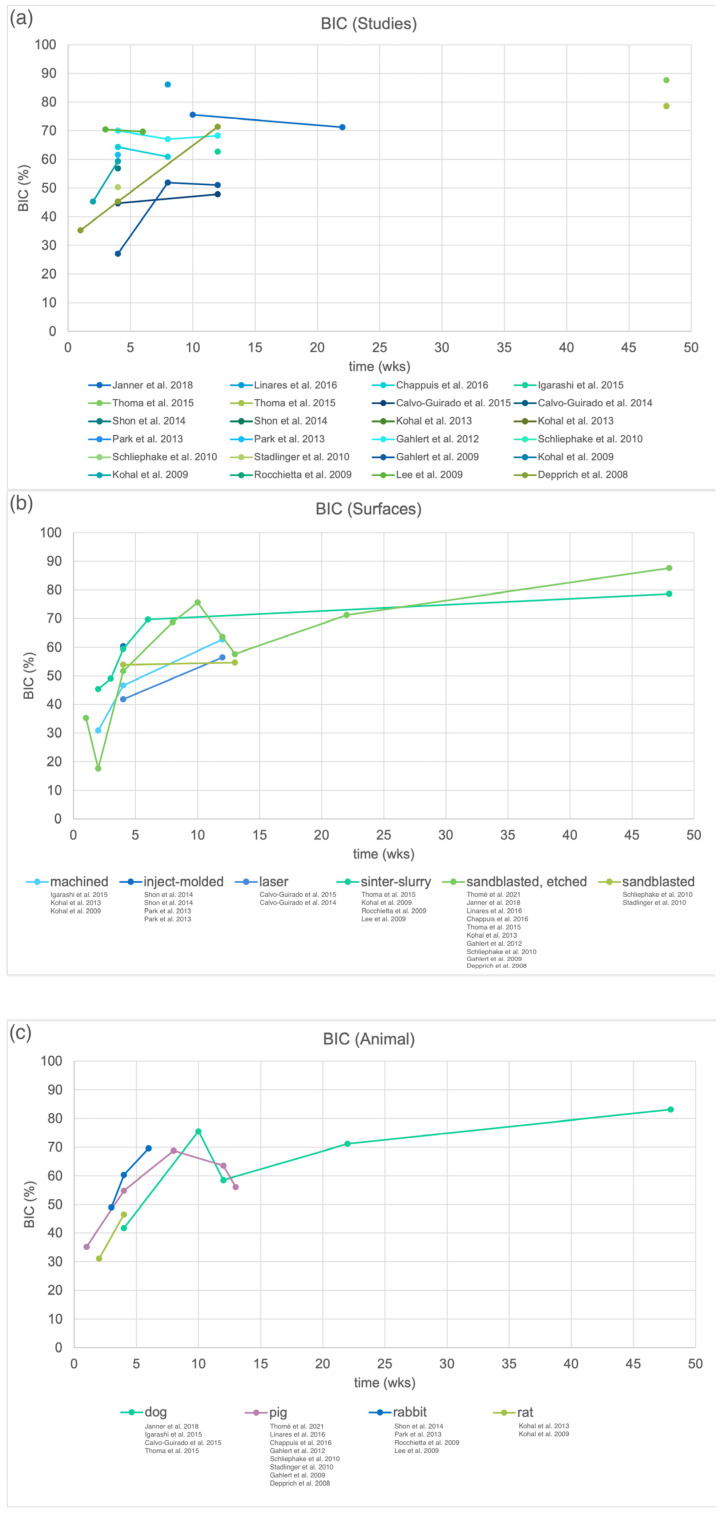
Bone-to-implant contact (BIC) around zirconia implants obtained in preclinical studies. (**a**) BIC of all included studies, (**b**) BIC pooled by surface treatment of zirconia implants and (**c**) BIC pooled by animal model.

The BIC at the respective intervention time for each study is displayed in Figure 3a. Similar surface treatments were pooled in Figure 3b. No clear preference of one surface treatment is observable. Figure 3c shows the BIC pooled by the animal model for zirconia implants. The rabbit and rat models display a short intervention time with a fast osseointegration; however, with the rabbit model, higher BIC values were reported. The BIC with the pig model decreased again after 8 weeks. For the dog model, osseointegration progressed slower and required observation periods of up to 48 weeks. 

**Figure 4:**
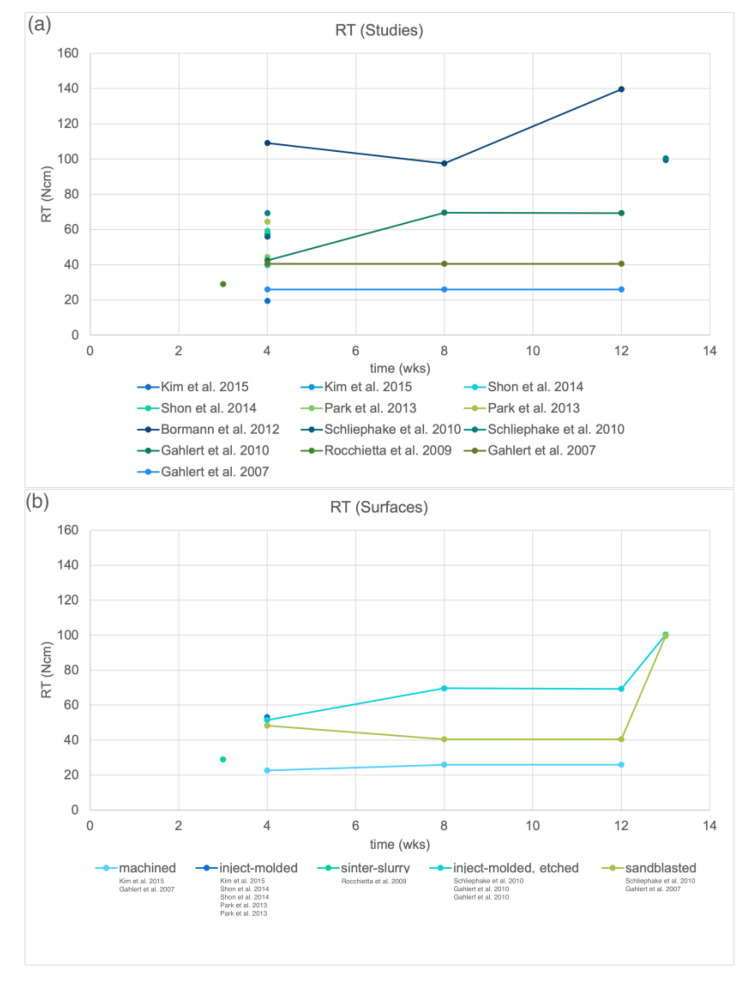

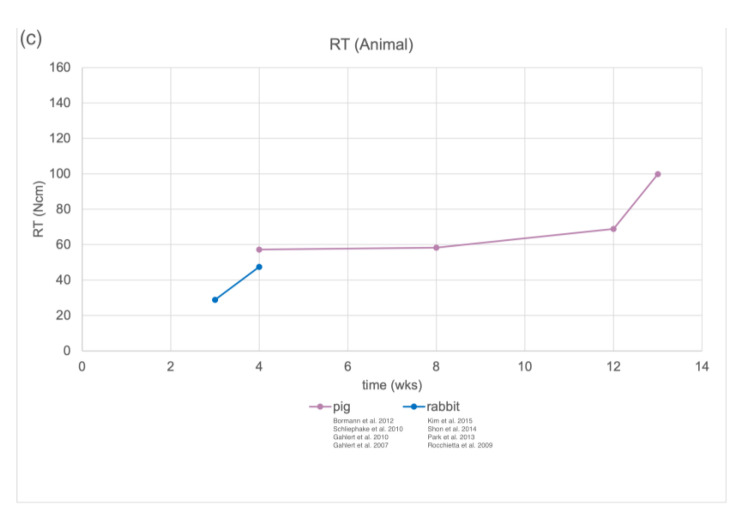
Removal torque (RT) around zirconia implants obtained in preclinical studies. (**a**) RT of all included studies, (**b**) RT pooled by surface treatment of zirconia implants and (**c**) RT pooled by animal model.

Figure 4a reveals the RT at the different intervention times for each included preclinical study. Similar surfaces were pooled in Figure 4b. For machined surfaces, a lower RT was reported than for modified surfaces that were sandblasted or etched. Studies comparing the RT of zirconia implants with different surfaces within the same set-up found higher values for rougher surfaces [53,61,67]. When the RT was pooled by the animal model in Figure 4c, the RT in the rabbit model strongly increased between 3 and 4 weeks. For the pig model, a slight increase was reported between 8 and 12 weeks that progressed further afterward.

Cell studies with osteoblastic cells comparing two differing zirconia implant surfaces are presented descriptively in Table 3 (n = 7). Six of the studies compared a micro-structured (sandblasted and subsequently etched [50,68,69,70,71] or sinter and slurry-modified [72]) surface with a machined surface. All studies showed that cell spreading and cytoskeletal formation were enhanced on machined compared with micro-structured surfaces [50,68,69,70,72]. Cell proliferation was either enhanced [72], similar [50] or lower [71] on machined surfaces compared with micro-structured surfaces. For gene expression, no conclusive results were observable as all studies investigated different time intervals, gene expression and cell lines.

## 4. Discussion

The purpose of this review was to analyze and correlate the findings for zirconia implants in clinical with preclinical and in vitro cell studies to evaluate the influence of the surface structure on the outcome. As only sandblasted, sinter and slurry-modified, as well as sandblasted and subsequently etched surfaces were investigated in the included clinical trials, only those findings could be compared with preclinical and in vitro cell studies. The best performance with the highest survival rates and the lowest MBL in the clinic were observed for sandblasted and subsequently etched surfaces, followed by sinter and slurry-modified and sandblasted surfaces. In preclinical studies, the BIC values reported for sandblasted and subsequently etched and sinter and slurry-modified surfaces seemed similar, deviating slightly at each intervention point. Osseointegration of sandblasted surfaces progressed slower; however, data were only available for two intervention time points. Unfortunately, none of these surfaces were compared with each other regarding the RT and in cell studies with osteoblasts.

The zirconia implants currently on the market with clinical studies included in this review are either one-piece Y-TZP implants that are sandblasted by Bredent [28,30], sandblasted and etched by Straumann and Vita [16,19] or ATZ implants with surfaces modified with sinter and slurry by Fairimplant [17]. Implants of Z-System [29,31] are still available, but surfaces were changed from sandblasted to laser-modified. The two-piece implant system of Ziterion with clinical studies available [18] was acquired by Sirona Dentsply but is at present not on the market. For the clinical performance of two-piece implants, only limited clinical data are currently available. Besides the study with Ziterion implants [18], another clinical trial was conducted with Zeramex ATZ implants reporting a survival rate of 83% after almost a 7-year follow-up [74]. Within the currently available systems, the highest survival rates were reported for one-piece zirconia implants with sandblasted and subsequently etched surfaces of 97.5 to 98.4% [16,19]. In addition, the MBL is promising with values below 1 mm after 3 and 5.5 years, respectively, for those studies that evaluated 102 implants in total. The results of implants modified with sinter and slurry achieved high survival rates in two different studies of 95.4% to 98.2% after 1 year [32,33] and 94.3% after 5 years [17], respectively. All three studies were conducted by one group. The MBL of the most recent study with 35 evaluated ATZ implants after 5 years (Ziraldent FR1, Metoxit) was within the range of sandblasted and subsequently etched implants, i.e., below 1 mm [17]. However, the MBL of Y-TZP implants by Nobel Biocare with the same surface showed an MBL of 1.3 mm after 1 year for implants restored with single crowns [33]. A higher MBL of almost 2 mm was reported for those implants restored with fixed dental prostheses (FDP) [32]. Follow-ups in both studies were only conducted up to one year. The restorative treatment (single crowns or FDPs) affected the MBL in all studies with sinter and slurry-modified surfaces [17,32], but not for sandblasted and subsequently etched zirconia implants [16]. Implants with sandblasted surfaces displayed lower survival rates, varying between 77.3 and 100% [18,28,29,30,31], confirmed by a tendency towards higher MBL values than for other implant surfaces.

Overall, the clinical outcome of zirconia implants irrespective of the surface can be considered promising confirming previous reviews [21,25,26,27], and all implant surfaces resulted in an MBL below the threshold value for clinical success of 2 mm [34]. In clinical studies, only the micro-structured implant surfaces are investigated. However, it has been found that clinical bone formation on zirconia implants occurs and remains stable up to 5 years on the endosseous sandblasted and subsequently etched, as well as on the polished transmucosal part [75]. The current review confirms findings of a review with titanium implants demonstrating that very good long-term results can be achieved with all types of endosseous implant surfaces (turned, titanium plasma sprayed, blasted, anodized, blasted and acid-etched) [76]. Hence, the surface structure of the endosseous part of implants may not be the decisive factor for osseointegration but might affect osseointegration speed. Such a hypothesis is, however, not supported by the available results.

In preclinical studies, no clear preference of one surface structure could be identified. Surprisingly, surfaces with lower surface roughness that were machined or injection-molded provided comparable BIC to modified surfaces. When smooth surfaces were directly compared with rougher surfaces within the same set-up, even higher [50], similar [51,52,53] or lower [54] BICs were measured. Hence, the current dogma for titanium implants, that an Sa value between 1 and 2 µm roughness promotes osseointegration [11], may not be applicable for zirconia implants. The surface roughness value Sa is mainly used to describe the roughness of implants. However, this value only provides information on the arithmetical mean roughness and the actual topography, while factors such as kurtosis and skewness that may impact osseointegration are not considered. As obtaining the surface roughness of dental implants was not performed in a standardized manner for the included studies, the authors refrained from using the surface roughness parameter Sa as a comparative factor in this review.

The RT values were lower for machined compared with rougher surfaces [53,61,67]; hence, a machined implant may be easier retrieved in the case of removal. However, the effect of a smooth surface on the long-term stability of the implant is currently not known.

As previously observed, BIC and RT were highly affected by the selected animal model [27]. Osseointegration occurred faster in the rabbit and rat models; hence, shorter investigation times seemed applicable. Higher BIC values can be expected from the rabbit model compared with the rat model for zirconia implants (Figure 3c). Larger animal models with dogs or pigs are commonly chosen, as they would provide an ossseointegration speed similar to the human species [77]. However, longer observation periods and higher costs are to be expected for those studies. Consequently, BIC and RT values of the different surfaces could only be truly evaluated within the same study set-up due to varying effects of the animal model, observation time, implant material and implant dimensions. Hence, before considering performing an animal study, those parameters should be as standardized as possible to retrieve a valuable outcome on the effect of surface structure.

Cell studies should be a prerequisite for conducting animal studies. Only one group considered comparing the outcome of their cell study with a preclinical model with rats using the same surfaces [50]. Machined surfaces were compared with sandblasted and subsequently etched surfaces. For cell proliferation and gene expression of human fetal osteoblast cells hFOB 1.19, no correlation between the outcome of cell and animal study could be determined between the two investigated surfaces. However, cytoskeletal organization and focal contact formation were faster on machined than on sandblasted and subsequently etched surfaces. In addition, the BIC in the rat model was significantly higher on the machined surface after 4 and 8 weeks [50]. The finding that cell spreading and cytoskeletal formation are enhanced on machined compared with micro-structured surfaces was confirmed by all other included studies with osteoblast cells [50,68,69,70,72].

Unfortunately, no clinical study with machined zirconia implants exists to further investigate the potential of this surface structuring method. Only the fact that radiological long-term data from a clinical study prove a tight contact between the polished zirconia transmucosal part and bone [75] supports the hypothesis that smooth surfaces might provide osseointegration in the long run. Smooth surfaces are of high interest to improve cleanability, increase implant quality and decrease production costs.

Although a great effort is made to conduct in vitro, preclinical and clinical studies, no correlation can be observed within the respective results, underlining the need for standardized procedures for animal and human studies, especially concerning ethics. Additional attention should be focused on choosing an appropriate control group for animal and human research having only one varying parameter from the test group. The tested materials and surfaces are to be properly characterized using scanning electron microscopy and more parameters of surface roughness than the arithmetical mean. In vitro cell and preclinical studies using the same implant surfaces should be mandatory prior to a clinical trial to validate the study designs. In addition, there is a strong need for guidelines for conducting clinical trials with implants and for the parameters to be obtained and reported.

## 5. Conclusions

The conclusion must be interpreted with care as the study designs of the included clinical, preclinical and cell studies are highly heterogenous and vary regarding the follow-up time and implant design. Within the limitations of this review that investigated the impact of surface structure of zirconia implants it can be concluded that:

- In clinical studies, the lowest MBL was reported for sandblasted and subsequently etched surfaces, followed by a sinter and slurry treatment and sandblasted surfaces;
- In preclinical studies analyzing BIC, no clear preference of one surface structure was observable. The RT was slightly higher for micro-structured than smooth surfaces. The BIC and RT values were highly influenced by the chosen animal model;
- All cell studies showed that cell spreading and cytoskeletal formation were enhanced on machined compared with micro-structured surfaces;
- No correlation was observed between the outcomes, underlining the need for standardized procedures for animal and human studies.

## Figures and Tables

**Table 1:** Overview of clinical trials with zirconia implants.

Author/Year	Publication	Implants (n)	Material	Surface Treatment	Type, Manufacturer	Follow-Up after Placement (yrs)	Survival (%)
Steyer et al. 2021	[28,35]	20 (11 evaluated)	Y-TZP	Sandblasted Sa 1.17 µm	WhiteSky, Bredent medical, Germany	11	80.0%
Kohal et al. 2020	[17,36,37]	53 (47 evaluated)	ATZ	sandblasted, sintering of ceramic slurry (Zircapore) Ra 1.8 µm	Ziraldent FR1, Metoxit, Switzerland (now FairWhite, Fairimplant, Germany)	5	94.3%
Koller et al. 2020	[18,38]	16 (14 evaluated)	Y-TZP	sandblasted	Vario z, Ziterion, Germany (now Sirona Dentsply)	7	93.3%
Balmer et al. 2020	[16,39,40]	71 (63 evaluated)	Y-TZP	sandblasted, etched, heat-treated (cer.face 14) Ra 1.2 µm	ceramic.implant, VITA, Germany	5.5	98.4%
Bormann et al. 2018	[19,41]	44 (39 evaluated)	Y-TZP	sandblasted, etched (ZLA) Sa 0.7 µm	Pure, Straumann, Switzerland	5	97.5%
Roehling et al. 2016	[29]	161 (125 evaluated)	Y-TZP	sandblasted	Z-Lock3, Z-Systems, Switzerland	5.9	77.3%
Kohal et al. 2013	[32]	56 (55 evaluated)	Y-TZP	porous sintered zirconia slurry (Zircapore) Sa 1.24 µm	ZiUnite, Nobel Biocare, Sweden	1	98.2%
Borgonovo et al. 2013	[30,42,43,44]	35 (28 evaluated)	Y-TZP	Sandblasted Sa 0.9–1 µm	WhiteSky, Bredent medical, Germany	4	100%
Kohal et al. 2012	[33]	66 (63 evaluated)	Y-TZP	porous sintered zirconia slurry (Zircapore) Sa 1.24 µm	ZiUnite, Nobel Biocare, Sweden	1	95.4%
Cannizaro et al. 2010	[31]	40 (40 evaluated)	Y-TZP	sandblasted	Z-Lock3, Z-Systems, Switzerland	1	87.5%

**Table 2:** Overview of preclinical studies that measured bone-to-implant contact or removal torque (mean with standard deviations) of zirconia implants.

Author/Year	Publication	Animal	Material	Surface Treatment	Manufacturer	Observation (wks)	BIC (%)	RT (Ncm)
Thomé et al. 2021	[55]	Pig n = 6	Y-TZP	Injection-molded, sandblasted, etched (ZLA) Sa 0.76 µm	Neodent Zi ceramic implant, Straumann, Switzerland	8	77.8 ± 6.9	-
Janner et al. 2018	[56]	Dog n = 5	Y-TZP	Sandblasted, etched	Straumann, Switzerland	10, 22	75.6 ± 6.3, 71.2 ± 7.0	-
Linares et al. 2016	[57]	Pig n = 6	Y-TZP	Sandblasted, etched	Straumann, Switzerland	8	86.2 ± 9.7	-
Chappuis et al. 2016	[58]	Pig n = 7	Y-TZP	Sandblasted, etched Sa 0.9 µm	Zerafil TZP, Dentalpoint, Switzerland	4, 8	64.4, 60.9	-
Igarashi et al. 2015	[59]	Dog n = 5	Y-TZP	Machined Ra 0.11 µm	TZ-3YS-E, Tosoh Corporation, Japan	12	62.7	-
Thoma et al. 2015	[49]	Dog n = 6	Y-TZP	Sandblasted, etched, heat-treated (cer.face 14)	VITA, Germany	48	87.7 ± 25.1	-
			ATZ	Sandblasted, sintering of ceramic slurry (Zircapore)	Metoxit, Switzerland		78.6 ± 17.3	
Calvo-Guirado et al. 2015	[60]	Dog n = 6	Y-TZP	Laser modification	Bredent, Germany	4, 12	44.7 ± 17.7, 47.9 ± 16.2	-
Kim et al. 2015	[61]	Rabbit n = 16	Y-TZP	Machined Sa 0.58 µm	Dentime, South Korea	4	-	19.4± 7.4
				Injection-molded Sa 1.67 µm	Cetatech, South Korea			57.6± 11.6
Calvo-Guirado et al. 2014	[62]	Dog n = 6	Y-TZP	Laser modification	Bredent, Germany	4, 12	38.9 ± 6.7, 65.0 ± 4.7	-
Shon et al. 2014	[53]	Rabbit n = 25	Y-TZP	Injection-molded Smooth Sa 0.54 µm	Cetatech, South Korea	4	58.3 ± 10.1	39.7 ± 11.7
				Injection-molded Rough Sa 1.98 µm			56.9 ± 13.0	59.2 ± 12.3
Kohal et al. 2013	[50]	Rat n = 56	Y-TZP	Sandblasted, etched, heat-treated (cer.face 14) Sa 0.95 µm	VITA, Germany	2, 4	17.6 ± 1.4, 33.5 ± 4.1	-
				Machined Sa 0.19 µm			30.9 ± 10.1, 46.6 ± 13.9	
Park et al. 2013	[52]	Rabbit n = 20	Y-TZP	Injection-molded Smooth Sa 0.53 µm	Chaorum, South Korea	4	61.6 ± 12.4	44.3 ± 8.4
				Injection-molded Rough Sa 2.00 µm			64.4 ± 11.5	64.4 ± 10.5
Gahlert et al. 2012	[45]	Pig n = 18	Y-TZP	Injection-molded, etched Sa 0.63 µm	Straumann, Switzerland	4, 8, 12	70.0 ± 14.5, 67.1 ± 21.1, 68.3 ± 22.8	-
Bormann et al. 2012	[46]						-	109.0 ± 24.2, 97.4 ± 29.3, 139.6 ± 56.6
Schliephake et al. 2010	[51]	Pig n = 12	Y-TZP	Sandblasted Sa 1.0 µm	Thommen Medical, Switzerland	4, 13	57.5 ± 14.3, 54.6 ± 17.6	55.9 ± 18.4, 99.4 ± 30.9
				Sandblasted, etched Sa 1.2 µm			69.3 ± 17.1, 57.6 ± 23.7	69.3 ± 17.1, 100.3 ± 47.0
Stadlinger et al. 2010	[63]	Pig n = 7	Y-TZP	Sandblasted Ra 1.0 µm	Bredent, Germany	4	50.3 ± 17.9	-
Gahlert et al. 2010	[47]	Pig n = 16	Y-TZP	Injection-molded, etched Sa 0.59 µm	Straumann, Switzerland	4, 8, 12	-	42.4 ± 15.1, 69.6 ± 25.1, 69.3 ± 24.2
Gahlert et al. 2009	[48]						27.1 ± 3.5, 51.9 ± 14.0, 51.1 ± 12.4	-
Kohal et al. 2009	[54]	Rat n = 28	Y-TZP	Machined Ra 0.13 µm	Metoxit, Switzerland	2, 4	30.9, 46.6	-
				Sintering of ceramic slurry Ra 0.36 µm			45.3, 59.4	
Rocchietta et al. 2009	[64]	Rabbit n = 18	Y-TZP	Sintering of ceramic slurry Sa 1.24 µm	Zi-Unite, Nobel Biocare, Sweden	3	27.5 ± 54.5	28.9 ± 8.7
Lee et al. 2009	[65]	Rabbit n = 20	Y-TZP	Sintering of ceramic slurry Ra 1.0 µm	Zi-Unite, Nobel Biocare, Sweden	3, 6	70.5 ± 3.1, 69.7 ± 5.7	-
Depprich et al. 2008	[66]	Pig n = 12	Y-TZP	Etched Sa 0.60 µm	Konus Dental Implants, Germany	1, 4, 12	35.3 ± 10.8, 45.3 ± 15.7, 71.4 ± 17.8	-
Gahlert et al. 2007	[67]	Pig n = 13	Y-TZP	Sandblasted Sa 0.56 µm	Metoxit, Switzerland Straumann, Switzerland	4, 8, 12		40.5
				Machined Sa 0.13 µm				25.9

**Table 3:** Overview of cell studies with osteoblast that compared two different zirconia implant surfaces by measuring cell spreading, cell proliferation or gene expression.

Author/Year	Publication	Cell Type	Material	Surface Treatment	Manufacturer	Cell Spreading	Cell Proliferation	Gene Expression
Rohr et al. 2020	[68]	MG-63	Y-TZP	Machined, polished, sandblasted, etched (cer.face 14)	Vita, Germany	Significantly higher for machined and polished surfaces than sandblasted, etched zirconia after 20 min and 24 h	-	No significant difference in gene expression of ALP, COL and OCN for all surfaces after 24 h and 3 d
Jung et al. 2020	[70]	hOB	Y-TZP	Machined, sandblasted, etched (ZLA)	Straumann, Switzerland	Enhanced cell spreading visualized with actin staining on machined surface compared with sandblasted, etched surface	-	Gene expression of ACTB and fibronectin upregulated and Vimentin, VCL and focal adhesion kinase and laminin downregulated of hOB cells on sandblasted, etched surface compared with machined surface after 7 and 14 d
Jung et al. 2018	[71]	hOB	Y-TZP	Machined, Sandblasted, etched (ZLA)	Straumann, Switzerland	-	Cell viability and proliferation higher on sandblasted, etched surface compared with machined surface after 14 d	Gene expression of osteoprotegerin upregulated, RUNX2 downregulated, osteopontin similar on sandblasted, etched surface compared with machined surface after 7 and 14 d
Delgado-Ruiz et al. 2016	[73]	hFOB 1.19	Y-TZP	Sandblasted, sandblasted, laser-modified	Bredent, Germany	Cell spreading comparable observed in SEM	Higher cell density on sandblasted, laser-modified surface than on sandblasted after 7 and 15 d	Higher alkaline phosphatase on sandblasted, laser-modified surface than on sandblasted surface after 7 and 15 d
Bergemann et al. 2015	[69]	hOB	Y-TZP	Machined, sandblasted, etched (cer.face 14)	Vita, Germany	Significantly higher for machined than sandblasted, etched zirconia after 24 h	-	No significant difference in gene expression of alkaline phosphatase, collagen for both surfaces after 24 h and 3 d, osteocalcin after 24 h similar and significantly higher for cer.face 14 after 3 d
Kohal et al. 2013	[50]	hFOB 1.19	Y-TZP	Machined, sandblasted, etched	Vita, Germany	Cytoskeletal organization and focal contact formation faster on machined than sandblasted, etched surface	Similar cell proliferation between 1 and 28 d on both surfaces	No conclusive results on gene expression of BMP7, collagen, integrins, proteoglycans and osteocalcin
Setzer et al. 2009	[72]	hFOB 1.19	ATZ	machined; porous sintered zirconia slurry (Zircapore),	Nobel Biocare, Sweden	Cell spreading and cytoskeleton formation enhanced on machined surface compared with Zircapore surface after 4 and 24 h	Proliferation higher on machined surface compared with Zircapore surface after 1, 3 and 7 d	No conclusive results on gene expression of a wide range of proliferation, maturation, mineralization and cell cycle genes

## Data Availability

Data are available on request from the authors.
